# Trends of Substance Use among Individuals with Cardiovascular Disease in the United States, 2015–2019

**DOI:** 10.3390/ijerph19010577

**Published:** 2022-01-05

**Authors:** Yeonwoo Kim, Sehun Oh, Paul J. Fadel, Christopher P. Salas-Wright, Michael G. Vaughn

**Affiliations:** 1 Department of Kinesiology, University of Texas at Arlington, Arlington, TX 76010, USA; paul.fadel@uta.edu; 2 College of Social Work, The Ohio State University, Columbus, OH 43210, USA; oh.570@osu.edu; 3 School of Social Work, Boston College, Chestnut Hill, MA 02467, USA; Christopher.Salas-Wright@bc.edu; 4 College for Public Health and Social Justice, Saint Louis University, St. Louis, MO 63104, USA; michael.vaughn@slu.edu

**Keywords:** substance use, cannabis use, cardiovascular disease

## Abstract

Despite the adverse effects of substance use on health among individuals with preexisting cardiovascular disease (CVD), little is known about trends and correlates for substance use among individuals with CVD. We examined trends of use in tobacco, alcohol, and cannabis among US adults with heart disease. Using nationally representative data from the 2015–2019 National Survey on Drug Use and Health (N = 7339), we conducted survey-adjusted logistic regression analyses to test the significance of trends in substance use while controlling for sociodemographic factors and related correlates. Results showed that the prevalence of cannabis use among adults with a heart condition significantly increased. Notably, the prevalence of cannabis use increased by 91% among non-Hispanic Whites, while the increasing trends were not present among other racial/ethnic groups. Our results also showed that increase in cannabis use was associated with easier access, lower disapproval, and risk perceptions of cannabis. Special attention is needed to raise awareness of the risk associated with cannabis use among individuals with CVD and the implementation of an early screening and treatment strategy among those with CVD.

## 1. Introduction

In the United States (U.S.), substance misuse is one of the major social concerns. Not only does the prevalence of tobacco and alcohol use remain high, but the prevalence of cannabis use has also increased steadily among general U.S. populations [1,2,3,4,5]. For instance, McCarthy found that current cannabis use among adults almost doubled from 7% in 2013 to 13% in 2016 [5]. The epidemiological literature suggested that the important changes in cannabis use are related to the legalization of medical and recreational cannabis, which led to changes in perceptions of cannabis use among general U.S. populations [6].

Despite the potential for therapeutic benefits of moderate use of certain substances (e.g., alcohol and cannabis) for pain, appetite, sleep, and other health problems [7,8,9], detrimental health effects have also been documented [10,11,12,13,14,15]. In particular, because substance use may increase heart rate and blood pressure via sympathetic activation, elevating myocardial oxygen demand, substance use has adverse effects on the cardiovascular system in the general population [16,17,18,19,20]. Furthermore, the adverse effects on cardiovascular events and pathologies can be critical for patients diagnosed with cardiovascular disease (CVD) [21,22]. Empirical evidence reports that substance use increases the risk of recurrent cardiovascular events and complications among those with preexisting CVD [22,23,24,25,26,27].

Although the adverse effect of substance use on cardiovascular health has been documented [21,22,23,24,25,26,27], only few studies have investigated the prevalence of substance use among those with CVD. One recent study [27] used the electronic health record of the University of California San Diego Healthcare Systems and showed that 15.2% of their patients with heart failure misused any of alcohol, cannabis, opioids, methamphetamines, cocaine, barbiturates, and phencyclidine. The study also reported that the annual incidence of any substance misuse among those with heart failure increased from 2006 to 2016. Another recent study [21] used data from the National Survey on Drug Use and Health (NSDUH), a nationally representative study, and found that 2.3% of adults who reported cannabis use had CVD, which is estimated to be two million Americans with CVD by applying sampling weights. Importantly, however, no studies have examined the trends in substance use among individuals with CVD using a nationally representative sample.

In addition, it is not clear what is driving or associated with the trends of substance use among those with CVD. Investigating what might have contributed to the changes in substance use is especially important for cannabis use because a growing number of epidemiological trend studies provide compelling evidence that adult cannabis use is on the rise. A few studies that investigated cannabis use among general U.S. populations indicate that recent changes in cannabis-specific contexts, such as increasing access to cannabis and a decline in perceived harm of cannabis use, were associated with the increases in cannabis use and use disorders [4,28,29,30].

The present study takes an initial step to investigate the secular trends of substance use among U.S. individuals with CVD by using a nationally representative survey data from 2015 to 2019. We focus on tobacco, alcohol, and cannabis, as these are three of the most commonly used substances among general populations [31,32]. In addition, we investigated racial/ethnic patterns, which is consistent with prior studies reporting racial/ethnic variations, such as higher rates of alcohol and tobacco use among White adults compared to their Black counterparts [33] and recent increases in cannabis use among non-White adults compared to White counterparts [34]. Lastly, given the evidence of increasing adult cannabis use and substantial changes in the legal and societal contexts of U.S. cannabis use [6,16,17,18,19,20], this study devotes special attention to cannabis-specific contexts. As a supplementary analysis, we examined how cannabis-specific contexts (i.e., accessibility to cannabis, risk perception of cannabis use, and disapproval of cannabis use) have evolved in recent years and are associated with the latest cannabis trends among adults with CVD. Knowledge gained in this study can inform behavioral health policy, educational efforts, and intervention development aimed at reducing the adverse effect of substance use on those with CVD.

## 2. Materials and Methods

### 2.1. Data

Data were derived from the 2015–2019 NSDUH, a cross-sectional study of a nationally representative sample of non-institutionalized individuals in the U.S. The NSDUH provides population estimates for an array of substance use and health-related outcomes. This study uses comparable estimates from the 2015–2019 NSDUH since the latest survey redesign in 2015 [35]. Our analytic sample was restricted to 7339 adults who reported having a heart condition or heart disease diagnosed by a doctor or other healthcare professional. More details about the NSDUH study design are described elsewhere [35]. Since this study used the deidentified, public-use NSDUH data, IRB approval was exempt.

### 2.2. Measures

We examined respondent’s past 30-day use (0 = no, 1 = yes) of tobacco (i.e., cigarette, cigar, pipe tobacco, and smokeless tobacco), alcohol, and cannabis (i.e., marijuana or hashish). The past 30-day use is considered as current use. We also included three measures of cannabis-specific contexts (i.e., difficult access to cannabis, perception of great risk of cannabis use, and disapproval of cannabis use) as factors of marijuana use. Difficult access to cannabis was coded as 0 = fairly/very easy and 1 = fairly/very difficult or probably impossible, in response to the question, “How difficult or easy would it be for you to get some marijuana, if you want some?” Perception of great risk of cannabis use was coded 0 = none/slight/moderate risk and 1 = great risk, in response to the question, “How much do people risk harming themselves physically and in other ways when they smoke marijuana once or twice a week?” Disapproval of cannabis was coded as 0 = neither approve nor disapprove and 1 = somewhat/strongly disapprove in response to the question, “How do you feel about adults trying marijuana or hashish once or twice?”.

Sociodemographic variables included age (18–25; 26–34; 35–64; 65+), sex (male; female), race/ethnicity (Black; Hispanic; White; Other), education (less than high school; high school; some college; college), employment status (employed; unemployed; not in labor force), household income (less than $20,000; $20,000–$39,999; $40,000–$74,999; $75,000+), marital status (married; widowed/divorced/separated; never married), any health insurance coverage status (yes; no), and urbanicity of residence (urban; rural). We also included severe psychological distress (yes; no) as covariates because high exposure to stressful events in the past year has been documented to be associated with elevated risk of tobacco, alcohol, and cannabis misuse to cope with psychological distress [36,37,38,39]. Respondents were asked how frequently respondents experienced (1) feeling nervous, (2) feeling hopeless, (3) feeling restless or fidgety, (4) feeling so sad or depressed that nothing could cheer them up, (5) feeling that everything was an effort, and (6) feeling down on themselves, no good, or worthless, based on the Likert scale (0 = none of the time, 1 = a little of the time, 2 = some of the time, 3 = most of the time, and 4 = all of the time). Respondents were coded as 1 = having severe psychological distress if the summed score was 13 points or above (otherwise, 0 = no).

### 2.3. Statistical Analysis

Analyses were conducted in several steps, in a similar way to the past literature [4,28,29]. First, we examined the weighted crude prevalence of current tobacco, alcohol, and cannabis use and cannabis-specific contexts (i.e., difficult access to cannabis use, perception of greater risk of cannabis use, and disapproval of cannabis use) by year among individuals with CVD. As an ancillary analysis, we compared the weighted crude prevalence of current tobacco, alcohol, and cannabis use between those with and without CVD. Second, tests of trends in substance use were conducted by including survey year as a continuous independent variable and each of substance use as an outcome measure in logistic regression models for the total sample and then racial/ethnic subgroups, consistent with the Centers for Disease Control and Prevention’s guidelines [40]. In Model 1, we included survey year as a continuous independent variable without any covariates. We then added sociodemographic factors, psychological distress, and urbanicity of residence as covariates to the Model 1. If survey year was significantly associated with cannabis use in Model 2, in order to investigate the associations between cannabis-specific contexts and current cannabis use, the trends in current cannabis use were further tested by including cannabis-specific contexts as covariates in Model 3. Lastly, we examined trends in cannabis-specific contexts using multivariate logistic regression. We included each of cannabis-specific factors as an outcome measure, survey year as a continuous independent variable, and sociodemographic factors as covariates in logistic regression models for the total sample and then racial/ethnic subgroups. All estimates were weighted to account for the NSDUH’s stratified cluster sample design as suggested by the Substance Abuse and Mental Health Data Archive [41].

## 3. Results

### 3.1. Descriptive Statistics

Descriptive statistics show that the prevalence of current substance use among individuals with CVD was 19% (for tobacco), 46% (for alcohol), and 6% (for cannabis). Those who used substances were younger, more likely to be males, unemployed, unmarried, and uninsured compared to non-users (see Table 1).

### 3.2. Trends in Substance Use

**Figure 1:**
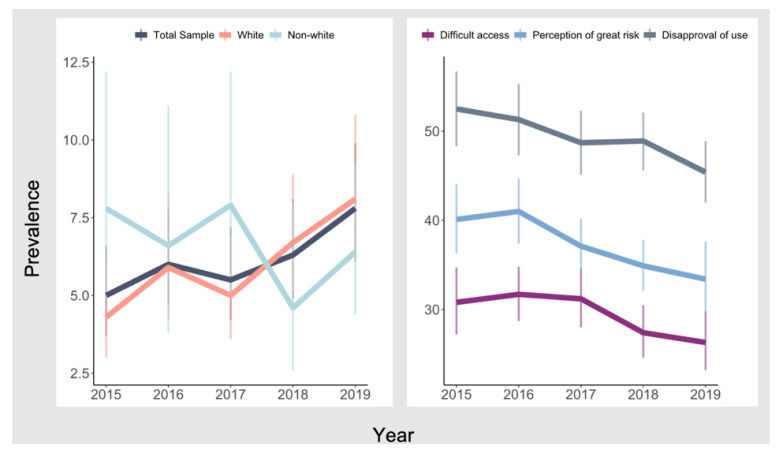
Trends in prevalence of past-month cannabis use and cannabis-specific factors among individuals with heart disease: pooled sample of NSDUH 2015–2019.

Table 2 presents trends in current tobacco, alcohol, and cannabis use among individuals with CVD. The bivariate model included only survey year as a continuous independent variable, and we then added covariates to the bivariate model. Results showed that there was no significant change in the annual rates of tobacco and alcohol use during the five-year period among the total sample (tobacco: adjusted OR = 0.99, 95% CI = 0.93–1.06; alcohol: adjusted OR = 1.00, 95% CI = 0.96–1.04) and all racial/ethnic subgroups. We observed increasing trends in current cannabis use while adjusting for sociodemographic factors (adjusted OR = 1.13, 95% CI = 1.02–1.25). Specifically, the rates of cannabis use have increased from 5.0% in 2015 to 7.8% in 2019, indicating a 56% increase during the five-year period. According to the subgroup analysis, significant increases in cannabis use were observed only among non-Hispanic Whites, from 4.3% in 2015 to 8.2% in 2019 (see Figure 1), indicating a 91% increase during the study period (adjusted OR = 1.17, 95% CI = 1.05–1.32). In addition, an ancillary analysis showed that the rates of cannabis use have increased among those without CVD from 8.7% in 2015 to 12.1% in 2019, indicating a 39% increase during the five-year period (see Appendix A Table A1).

### 3.3. Trends in Cannabis-Specific Contexts

Table 3 presents trends in cannabis-specific contexts from 2015 through 2019. The bivariate model included only survey year as a continuous independent variable, and we then added sociodemographic factors as covariates to the bivariate model. During the study period, notable decreases were observed in difficult access to cannabis (30.8% in 2015 to 26.3% in 2019), perception of great risk of cannabis use (from 40.1% in 2015 to 33.4% in 2019), and disapproval of cannabis use (from 52.5% in 2015 to 45.4% in 2019) among the whole sample (see Figure 1). When stratifying respondents by race/ethnicity, we found significant decreases in difficult access to cannabis (adjusted OR = 0.92, 95% CI = 0.86–0.98), perception of great risk of cannabis use (adjusted OR = 0.91, 95% CI = 0.86–0.96), and disapproval of cannabis use (adjusted OR = 0.93, 95% CI = 0.88–0.98) for non-Hispanic Whites.

### 3.4. Associations between Cannabis-Specific Contexts and Cannabis Use Trends

Table 4 shows results from the tests of trends in cannabis use among individuals with CVD while accounting for cannabis-specific contexts. We first included sociodemographic factors, severe psychological distress, and urbanicity of residence as covariates and then added each of the cannabis-specific factors as additional covariates to the model. Lastly, we included all three cannabis-specific factors in the full model. The associations between survey year and cannabis use ceased to be significant for models including any of three cannabis-specific contextual factors. For non-Hispanic Whites, when all three cannabis-specific contextual factors were included in the models, the magnitude of the associations between survey year and cannabis use became insignificant (adjusted OR = 1.10, 95% CI = 0.98–1.23). Full results of the analyses were presented in Appendix A Table A2, Table A3 and Table A4.

## 4. Discussion

CVD is one of the leading causes of mortality and disability in the U.S. In this analysis, we observed that a high proportion of U.S. adults with a heart disease use tobacco, alcohol, and/or cannabis (tobacco user: 19%, alcohol user: 46%, cannabis user: 6%). The statistics raise health concerns given the adverse effect of substance use on recurrent cardiovascular events and complications for those with preexisting CVD [22,23,24,25,26,27]. Those who use tobacco, alcohol, and/or cannabis are younger and more likely to be males, unemployed, unmarried, and uninsured compared to non-users. The sociodemographic differences are consistent with previous research [27].

In addition, our results showed that substance use trends among individuals with CVD varied by types of substances and race/ethnicity. While there were no notable changes in the prevalence of tobacco use and alcohol use from 2015 through 2019, cannabis use increased by 56% over the study period, primarily driven by the increased use among non-Hispanic Whites. In contrast to non-significant changes in cannabis use among other racial/ethnic groups, the rates among non-Hispanic Whites with CVD increased steadily from 4.3% in 2015 to 8.2% in 2019, indicating an 91% increase. These findings are interesting given that the results of our ancillary analysis showed that among those without CVD, both Whites and non-Whites had a significant increase in rates of cannabis use, which is consistent with past study examining cannabis use in the general population [34]. In addition, the magnitude of increase in cannabis use among Whites with CVD was higher than that of Whites without CVD observed in our ancillary analysis (44% increase among Whites without CVD) and reported in Han and Palamar’s study (43% increase among Whites in the general population) [34]. The findings imply that cannabis use trends among individuals with CVD may be different from patterns seen among those without CVD and that non-Hispanic Whites may be a primary group of focus for intervention.

Cannabis-specific contexts have significantly changed during the five-year period. We found significant decreases in limited access to cannabis, strong disapproval of cannabis use, and perceptions of cannabis use as a great risk to one’s health and well-being. In addition, increase in cannabis use was associated with the coinciding changes in cannabis-specific contexts (i.e., easier access, lower risk perception, and greater approval of cannabis use). This is consistent with prior research on cannabis use and other risky behaviors among the general population [4,28,29,30]. This implies that education and policy approaches to address these cannabis-specific contexts need to be considered as key preventative measures for cannabis use among individuals with CVD [21,22,26]. Health professionals need continuing education on recent changes in cannabis-specific contexts, such as accessibility, risk perception, and disapproval. While smoking is commonly asked by health professionals, these queries may be specified to distinguish smoking marijuana from cigarettes for effective screening. Lastly, given the association between the increasing cannabis use and decreasing perception of risk from using cannabis use once or twice a week, individuals with CVD need to be advised of the potential risk of cannabis when choosing to use cannabis.

We observed that those with severe psychological distress in the past year were more likely to use tobacco or cannabis than those without severe psychological distress after adjusting for sociodemographic factors and cannabis-specific contexts (tobacco: adjusted OR = 1.47, *p* < 0.001; cannabis: adjusted OR = 1.79, *p* < 0.001) (see Appendix A Table A2 and Table A3). The results parallel past research highlighting the role of psychological distress in substance use among the general population [36,37,38,39]. The results imply that one strategy to reduce substance use among individuals with CVD would be to monitor psychological distress levels among patients with CVD and assist with their cognitive and behavioral strategies to cope with stress.

The present study has several limitations to note. First, NSDUH data are cross-sectional, thereby limiting the causal interpretation of the relationships between cannabis-specific contexts and drug use. We were also unable to examine how trends in substance use changed before and after CVD diagnosis because the NSDUH does not provide longitudinal information on individuals’ substance use patterns over time. Second, since the latest survey redesign in 2015, we could not examine substance use trends before 2015. Third, all data are respondents’ self-reports, which could have resulted in an over- or under-estimation of sensitive behaviors, such as substance use. As having a heart disease diagnosed by a doctor and other healthcare professional was also self-reported, there is the potential for recall bias. Lastly, although urbanicity of residence was controlled in the model, we were unable to include other geographical characteristics such as state-level cultural and policy perspectives related to substance use. Future research needs to control geographical factors related to patterns in substance use.

## 5. Conclusions

Despite the aforementioned limitations, this study provides alarming statistics about substance use along with increasing cannabis use among those with CVD. The statistics inform for immediate actions to reduce the prevalence of substance among those with CVD. In addition, importantly, we see notable changes in cannabis-specific accessibility, risk perception, and disapproval, which may have been driving increases in cannabis use among individuals with CVD, particularly non-Hispanic Whites. The finding suggests the importance of raising awareness regarding the risks associated with cannabis use. Educating those with CVD on the potential negative health effect of substances would be warranted. Future research needs to examine the prevalence of cannabis use by forms, amount, and medical or non-medical purposes among individuals with CVD, which can inform policy, educational efforts, and intervention development. In particular, with decreased risk perceptions of cannabis, educating on the amounts of cannabis concentrate in different forms such as pastes, gummies, and e-vaping devices may be a preventative strategy to reduce unintentional ingestion of cannabis. In addition, longitudinal research is warranted to better understand how patterns in substance use change before and after CVD diagnosis. Lastly, a future investigation of the extent of the detrimental effect of substance use on those with CVD and its physiological mechanism(s) may help reduce recurrent cardiovascular events and complications.

## Figures and Tables

**Table 1:** Sample characteristics and prevalence of current substance use among adults with heart disease: pooled sample of NSDUH 2015–2019.

Characteristics	Unweighted Frequency (%)	Weighted % (95% Confidence Interval)		
		Tobacco	Alcohol	Cannabis
Total Sample	7339 (100.0)	19.2 (18.0–20.5)	46.1 (44.5–47.7)	6.2 (5.5–6.9)
Gender				
Men	3403 (46.4)	21.2 (19.7–22.7)	51.8 (49.7–53.9)	7.0 (6.1–8.0)
Women	3936 (53.6)	17.1 (15.6–18.8)	40.1 (38.0–42.2)	5.3 (4.4–6.4)
Age				
18–25	843 (11.5)	30.9 (27.0–35.1)	58.4 (53.3–63.3)	20.4 (16.6–24.9)
26–34	579 (7.9)	36.8 (31.9–42.1)	57.1 (52.8–61.4)	19.9 (15.9–24.5)
35–64	3114 (42.4)	29.0 (26.8–31.3)	49.9 (47.4–52.5)	8.6 (7.5–9.8)
65+	2803 (38.2)	10.2 (8.8–11.9)	41.9 (39.6–44.2)	2.8 (2.0–3.8)
Race/ethnicity				
Black	663 (9.0)	23.9 (20.3–28.0)	39.6 (34.9–44.6)	7.3 (5.2–10.3)
Hispanic	516 (7.0)	18.8 (14.3–24.4)	36.6 (29.9–43.8)	5.9 (4.0–8.6)
Other	492 (6.7)	22.9 (18.3–28.2)	39.2 (33.0–45.7)	6.3 (4.5–8.8)
White	5668 (77.2)	18.9 (17.2–20.0)	47.9 (46.2–49.6)	6.1 (5.3–7.0)
Education				
Less than high school	948 (12.9)	30.2 (26.3–34.3)	24.3 (21.1–27.7)	5.1 (3.8–6.9)
High school graduate	1989 (27.1)	23.2 (20.9–25.7)	35.5 (32.5–38.6)	6.0 (4.8–7.4)
Some college	2446 (33.3)	20.4 (18.4–22.6)	49.6 (46.8–52.4)	7.3 (5.9–8.9)
College graduate	1956 (26.7)	9.6 (8.1–11.4)	61.3 (58.1–64.4)	5.7 (4.4–7.3)
Employment				
Employed	3918 (43.6)	20.8 (18.5–23.2)	59.3 (57.1–61.5)	7.6 (6.4–9.1)
Unemployed	215 (2.9)	43.1 (32.4–54.5)	49.0 (39.2–58.8)	21.4 (12.8–33.7)
Not in labor force	3926 (53.5)	17.6 (16.0–19.3)	38.5 (36.6–40.5)	4.9 (4.2–5.8)
Household income				
<$20,000	1544 (21.0)	30.9 (27.7–24.3)	28.1 (25.1–31.4)	8.8 (6.7–11.4)
$20,000–39,999	1722 (23.5)	22.9 (20.6–25.3)	33.5 (30.3–36.7)	6.3 (5.0–7.9)
$40,000–74,999	1927 (26.3)	17.7 (15.4–20.2)	47.0 (44.1–49.9)	5.3 (4.3–6.7)
$75,000+	2146 (29.2)	11.6 (9.9–13.5)	63.8 (61.2–66.3)	5.4 (4.2–6.9)
Marital status				
Married	3655 (49.8)	14.9 (13.4–16.5)	51.5 (49.4–53.6)	4.4 (3.6–5.5)
Widowed/divorced/separated	2145 (29.2)	22.9 (21.1–24.9)	37.1 (34.6–39.8)	6.3 (5.2–7.6)
Never married	1539 (21.0)	30.0 (26.5–33.8)	45.0 (41.0–49.1)	14.5 (12.1–17.3)
Health insurance coverage				
Yes	6991 (95.3)	18.5 (17.3–19.7)	46.1 (44.4–47.7)	5.8 (5.1–6.6)
No	348 (10.7)	41.5 (33.4–50.2)	47.4 (38.9–56.0)	16.6 (10.9–24.5)
Urbanicity of residence				
Rural	782 (10.7)	24.8 (20.7–29.3)	38.0 (33.7–42.6)	4.0 (2.7–5.8)
Urban	6557 (89.3)	18.7 (17.5–20.1)	46.8 (45.0–48.5)	6.4 (5.6–7.2)
Survey Year				
2015	1420 (19.4)	20.1 (17.2–23.3)	46.1 (42.7–49.5)	5.0 (3.7–6.6)
2016	1397 (19.0)	19.8 (17.0–22.9)	45.9 (42.3–49.5)	6.0 (4.7–7.8)
2017	1462 (19.9)	18.4 (16.0–21.1)	46.2 (42.3–50.1)	5.5 (4.2–7.2)
2018	1564 (21.3)	19.8 (17.1–22.8)	46.2 (42.1–50.3)	6.3 (4.9–8.1)
2019	1496 (20.4)	18.1 (16.0–20.3)	46.2 (43.0–49.4)	7.8 (6.1–9.9)

**Table 2:** Test of trends in tobacco, alcohol, and cannabis use among adults with heart disease: pooled sample of NSDUH 2015–2019.

	All Individuals with a Heart Disease		Racial/Ethnic Subgroups							
			Black		Hispanic		Other		White	
	OR	95% CI	OR	95% CI	OR	95% CI	OR	95% CI	OR	95% CI
**Tobacco Use**										
Unadjusted (Bivariate)	0.97	0.92–1.03	1.05	0.90–1.22	1.01	0.83–1.24	0.87	0.68–1.10	0.97	0.91–1.04
Adjusted for Covariates	0.99	0.93–1.06	1.12	0.94–1.32	1.04	0.86–1.26	0.91	0.70–1.18	0.97	0.90–1.05
**Alcohol Use**										
Unadjusted (Bivariate)	1.00	0.96–1.05	1.01	0.89–1.16	0.89	0.74–1.07	1.08	0.84–1.39	1.01	0.95–1.06
Adjusted for Covariates	1.00	0.96–1.04	1.01	0.89–1.15	0.87	0.73–1.05	1.18	0.91–1.54	1.00	0.95–1.05
**Cannabis Use**										
Unadjusted (Bivariate)	1.11	1.01–1.23	0.99	0.77–1.26	0.83	0.61–1.13	0.96	0.76–1.22	1.16	1.04–1.30
Adjusted for Covariates	1.13	1.02–1.25	1.01	0.75–1.37	0.84	0.63–1.11	1.08	0.81–1.43	1.17	1.05–1.31

Note. The tests of trends were conducted using the pooled sample. Covariates include age, sex, education, employment status, household income, marital status, any health insurance coverage status, urbanicity of residence, year, and severe psychological distress. OR indicates odds ratios, and CI indicates 95% confidence intervals.

**Table 3:** Test of trends in cannabis-specific contexts among adults with heart disease: pooled sample of NSDUH 2015–2019.

	All Individuals with a Heart Disease		Racial/Ethnic Subgroups							
			Black		Hispanic		Other		White	
	OR	95% CI	OR	95% CI	OR	95% CI	OR	95% CI	OR	95% CI
**Cannabis-Specific Factors**										
Difficult Access ^1^										
Unadjusted (Bivariate)	0.94	0.89–0.99	0.95	0.80–1.13	1.14	0.92–1.41	0.85	0.66–1.08	0.92	0.87–0.98
Adjusted for Sociodemographic Factors	0.93	0.87–0.98	0.96	0.80–1.14	1.14	0.90–1.44	0.70	0.54–0.91	0.92	0.86–0.98
Perception of Great Risk ^2^										
Unadjusted (Bivariate)	0.92	0.87–0.97	0.97	0.83–1.13	0.91	0.77–1.08	0.88	0.69–1.12	0.92	0.87–0.97
Adjusted for Sociodemographic Factors	0.91	0.86–0.96	0.96	0.80–1.15	0.86	0.72–1.02	0.80	0.63–1.03	0.91	0.86–0.96
Disapproval ^3^										
Unadjusted (Bivariate)	0.94	0.89–0.99	0.94	0.80–1.10	1.00	0.81–1.25	0.96	0.81–1.14	0.93	0.88–0.98
Adjusted for Sociodemographic Factors	0.93	0.88–0.98	0.93	0.79–1.10	1.00	0.82–1.21	0.85	0.68–1.06	0.93	0.88–0.98

Note. The tests of trends were conducted using the pooled sample. Sociodemographic factors include age, sex, education, employment status, household income, marital status, any health insurance coverage status, urbanicity of residence, and year. OR indicates odds ratios, and CI indicates confidence intervals. ^1^ Difficult access to cannabis (0 = fairly/very difficult or probably impossible, 1 = fairly/very easy) was measured based on the question, “How difficult or easy would it be for you to get some marijuana, if you want some?” ^2^ Perception of great risk of cannabis (0 = none/slight/moderate risk, 1 = great risk) was measured based on the question, “How much do people risk harming themselves physically and in other ways when they smoke marijuana once or twice a week?” ^3^ Disapproval of cannabis (0 = neither approve nor disapprove, 1 = somewhat/strongly disapprove) was measured based on the question, “How do you feel about adults trying marijuana or hashish once or twice?”.

**Table 4:** Test of trends in cannabis use among adults with heart disease when cannabis-specific contexts of cannabis use were adjusted: pooled sample of NSDUH 2015–2019.

	All Individuals with a Heart Disease		Racial/Ethnic Subgroups							
			Black		Hispanic		Other		White	
	OR	95% CI	OR	95% CI	OR	95% CI	OR	95% CI	OR	95% CI
**Cannabis Use**										
Adjusted for Covariates	1.13	1.02–1.25	1.01	0.74–1.38	0.84	0.63–1.11	1.08	0.81–1.44	1.17	1.05–1.32
Additional Adjustments for Risk Factors										
Difficult Access ^1^	1.10	1.00–1.22	1.00	0.73–1.38	0.89	0.68–1.17	0.99	0.72–1.35	1.15	1.03–1.29
Perception of Great Risk ^2^	1.10	0.99–1.21	1.03	0.74–1.42	0.78	0.59–1.04	1.07	0.80–1.44	1.14	1.02–1.28
Disapproval of Use ^3^	1.09	0.99–1.20	0.93	0.68–1.27	0.85	0.60–1.20	1.01	0.73–1.39	1.14	1.02–1.28
Full Modell ^4^	1.06	0.96–1.16	0.97	0.70–1.34	0.78	0.55–1.12	0.99	0.70–1.39	1.10	0.98–1.23

Note. The tests of trends were conducted using the pooled sample. Covariates include age, sex, education, employment status, household income, marital status, any health insurance coverage status, urbanicity of residence, year, and severe psychological distress. OR indicates odds ratios, and CI indicates confidence intervals. ^1^ Difficult access to cannabis (0 = fairly/very difficult or probably impossible, 1 = fairly/very easy) was measured based on the question, “How difficult or easy would it be for you to get some marijuana, if you want some?” ^2^ Perception of great risk of cannabis (0 = none/slight/moderate risk, 1 = great risk) was measured based on the question, “How much do people risk harming themselves physically and in other ways when they smoke marijuana once or twice a week?” ^3^ Disapproval of cannabis (0 = neither approve nor disapprove, 1 = somewhat/strongly disapprove) was measured based on the question, “How do you feel about adults trying marijuana or hashish once or twice?” ^4^ The full model was adjusted for sociodemographic factors, severe psychological distress, and three cannabis-specific factors.

## Data Availability

The data presented in this study are available for access by the general population through the website of Substance Abuse and Mental Health Data Archive.

## Appendix A

**Table A1:** Test of trends in tobacco, alcohol, and cannabis use among adults with and without heart disease by year.

	Individuals with a Heart Disease						All Individuals without a Heart Disease					
	2015	2016	2017	2018	2019	Change Rate from 2015 to 2019	2015	2016	2017	2018	2019	Change Rate from 2015 to 2019
**Tobacco Use**												
Total Sample	20.1	19.8	18.4	19.8	18.1	−10.0%	26.1	25.8	24.4	23.4	23.2	−11.1%
White	19.5	19.0	18.0	19.4	17.1	−12.3%	28.1	28.3	26.6	25.8	25.5	−9.3%
Non-White	22.5	23.0	20.7	21.7	22.0	−2.2%	22.5	21.4	20.7	19.4	19.4	−13.8%
**Alcohol Use**												
Total Sample	46.1	45.9	46.2	46.2	46.2	0.2%	56.5	55.9	56.7	55.8	55.5	−1.8%
White	48.1	46.9	47.8	48.1	48.4	0.6%	61.7	61.5	61.8	61.2	60.8	−1.5%
Non-White	37.2	41.7	38.5	37.7	37.4	0.5%	47.3	45.9	48.0	46.8	46.9	−0.8%
**Cannabis Use**												
Total Sample	5.0	6.0	5.5	6.3	7.8	56.0%	8.7	9.2	10.0	10.7	12.1	39.1%
White	4.3	5.9	5.0	6.7	8.2	90.7%	8.8	9.3	10.4	10.8	12.7	44.3%
Non-White	7.8	6.6	7.9	4.6	6.4	−17.9%	8.4	9.1	9.4	10.6	11.3	34.5%

**Table A2:** Test of trends in tobacco and alcohol use among adults with heart disease: pooled sample of NSDUH 2015–2019.

	Tobacco Use				Alcohol Use			
	Unadjusted Model		Adjusted Model		Unadjusted Model		Adjusted Model	
	OR	95% CI	OR	95% CI	OR	95% CI	OR	95% CI
Survey Year	0.97	0.92–1.03	0.99	0.93–1.06	1.00	0.96–1.05	1.00	0.96–1.04
Gender								
Men			…				…	
Women			0.56	0.48–0.64			0.70	0.63–0.79
Age								
18–25			1.74	1.31–2.31			0.75	0.53–1.05
26–34			1.19	0.90–1.57			0.51	0.37–0.71
35–64			0.31	0.22–0.45			0.43	0.30–0.61
65+			…				…	
Race/ethnicity								
Black			0.80	0.62–1.03			0.90	0.72–1.14
Hispanic			0.53	0.36–0.78			0.71	0.51–0.99
Other			1.04	0.76–1.40			0.65	0.49–0.85
White			…				…	
Education								
Less than high school			3.11	2.31–4.20			0.35	0.28–0.45
High school graduate			2.18	1.71–2.78			0.50	0.41–0.62
Some college			1.85	1.43–2.40			0.79	0.66–0.94
College graduate			…				…	
Employment								
Employed			…				…	
Unemployed			1.79	0.89–3.58			0.95	0.59–1.53
Not in labor force			0.93	0.71–1.21			0.67	0.58–0.77
Household income								
<$20,000			2.36	1.72–3.25			0.41	0.32–0.52
$20,000–39,999			1.93	1.47–2.54			0.44	0.38–0.51
$40,000–74,999			1.61	1.25–2.09			0.63	0.53–0.75
$75,000+			…				…	
Marital status								
Married			…				…	
Widowed/divorced/separated			1.53	1.27–1.85			0.96	0.84–1.10
Never married			1.22	0.95–1.58			0.81	0.62–1.05
Health insurance coverage								
Yes			…				…	
No			1.23	0.90–1.70			1.37	0.96–1.94
Severe psychological distress								
Yes			1.47	1.20–1.79			0.99	0.83–1.19
No			…				…	
Urbanicity of residence								
Rural			1.18	0.91–1.54			0.91	0.74–1.12
Urban			…				…	

Note. The tests of trends were conducted using the pooled sample. OR indicates odds ratios, and CI indicates 95% confidence intervals.

**Table A3:** Test of trends in cannabis use among adults with heart disease: pooled sample of NSDUH 2015–2019.

	Model 1(Unadjusted)		Model 2		Model 3		Model 4		Model 5		Model 6	
	OR	95% CI	OR	95% CI	OR	95% CI	OR	95% CI	OR	95% CI	OR	95% CI
Survey Year	1.11	1.01–1.26	1.13	1.02–1.25	1.10	1.00–1.22	1.10	0.99–1.21	1.09	0.99–1.20	1.06	0.96–1.16
Gender												
Men			…		…		…		…		…	
Women			0.57	0.45–0.73	0.61	0.47–0.78	0.64	0.50–0.83	0.61	0.47–0.78	0.69	0.52–0.92
Age												
18–25			1.16	0.80–1.68	1.19	0.83–1.70	1.13	0.78–1.63	1.00	0.68–1.47	1.02	0.70–1.49
26–34			0.55	0.37–0.81	0.61	0.41–0.90	0.62	0.42–0.91	0.57	0.38–0.85	0.67	0.45–0.99
35–64			0.18	0.10–0.31	0.26	0.15–0.43	0.26	0.15–0.43	0.27	0.16–0.47	0.43	0.26–0.73
65+			…		…		…		…		…	
Race/ethnicity												
Black			0.83	0.53–1.28	0.83	0.53–1.29	0.94	0.61–1.46	0.88	0.55–1.42	0.95	0.59–1.52
Hispanic			0.60	0.38–0.95	0.70	0.44–1.12	0.75	0.47–1.21	0.68	0.41–1.13	0.92	0.56–1.51
Other			0.81	0.56–1.16	0.94	0.63–1.39	0.86	0.59–1.25	0.92	0.60–1.41	1.12	0.70–1.78
White			…		…		…		…		…	
Education												
Less than high school			0.66	0.39–1.12	0.76	0.44–1.29	0.80	0.46–1.40	0.74	0.42–1.31	0.95	0.53–1.69
High school graduate			0.81	0.55–1.19	0.87	0.59–1.26	0.90	0.60–1.34	0.83	0.56–1.24	0.95	0.65–1.38
Some college			0.99	0.67–1.48	0.99	0.67–1.46	1.05	0.70–1.57	1.02	0.67–1.55	1.06	0.70–1.61
College graduate			…		…		…		…		…	
Employment												
Employed			…		…		…		…		…	
Unemployed			2.17	1.07–4.41	2.41	1.23–4.73	1.94	1.00–3.77	2.22	1.05–4.72	2.21	1.15–4.26
Not in labor force			1.01	0.74–1.37	1.06	0.79–1.41	1.03	0.75–1.42	1.02	0.74–1.41	1.10	0.80–1.52
Household income												
<$20,000			1.49	0.86–2.58	1.59	0.90–2.83	1.60	0.91–2.80	1.65	0.94–2.87	1.70	0.93–3.10
$20,000–39,99			1.22	0.86–1.74	1.29	0.91–1.84	1.33	0.94–1.90	1.39	0.95–2.03	1.48	1.01–2.16
$40,000–74,999			1.04	0.71–1.52	1.07	0.74–1.55	1.05	0.72–1.53	1.13	0.77–1.65	1.13	0.78–1.64
$75,000+			…		…		…		…		…	
Marital status												
Married			…		…		…		…		…	
Widowed/divorced/separated			1.58	1.12–2.25	1.57	1.10–2.25	1.54	1.08–2.20	1.42	0.99–2.04	1.47	1.01–2.14
Never married			1.69	1.14–2.52	1.61	1.07–2.41	1.62	1.07–2.45	1.56	1.02–2.39	1.49	0.96–2.32
Health insurance coverage												
Yes			…		…		…		…		…	
No			1.56	0.93–2.60	1.58	0.93–2.66	1.50	0.91–2.46	1.58	0.95–2.63	1.68	1.00–2.83
Severe psychological distress												
Yes			1.79	1.37–2.33	1.69	1.29–2.20	1.61	1.21–2.14	1.56	1.19–2.03	1.47	1.11–1.94
No			…		…		…		…		…	
Urbanicity of residence												
Rural			0.60	0.38–0.94	0.54	0.35–0.86	0.61	0.39–0.94	0.62	0.39–0.99	0.58	0.37–0.91
Urban			…		…		…		…		…	
Difficult access to cannabis												
Yes					0.06	0.02–0.14					0.14	0.06–0.35
No					…						…	
Perception of risk of cannabis												
Yes							0.06	0.02–1.14			0.12	0.06–0.21
No							…				…	
Disapproval of cannabis use												
Yes									0.07	0.04–0.12	0.08	0.03–0.21
No									…		…	

Note. The tests of trends were conducted using the pooled sample. Model 1 included only survey year as a continuous variable; Model 2 included age, sex, race/ethnicity, education, employment status, household income, marital status, any health insurance coverage status, urbanicity of residence, year, and severe psychological distress. Model 3 added difficult access to cannabis to Model 2, Model 4 added perception of great risk of cannabis use to Model 2, and Model 5 added disapproval of cannabis use to Model 2. Model 6 added three cannabis-specific factors to Model 2. OR indicates odds ratios, and CI indicates 95% confidence intervals.

**Table A4:** Test of trends in cannabis use among non-Hispanic Whites with heart disease: pooled sample of NSDUH 2015–2019.

	Model 1 (Unadjusted)		Model 2		Model 3		Model 4		Model 5		Model 6	
	OR	95% CI	OR	95% CI	OR	95% CI	OR	95% CI	OR	95% CI	OR	95% CI
Survey Year	1.16	1.04–1.30	1.17	1.05–1.32	1.15	1.03–1.29	1.14	1.02–1.28	1.14	1.02–1.28	1.1	0.98–1.23
Gender												
Men			…		…		…		…		…	
Women			0.57	0.45–0.74	0.62	0.48–0.81	0.65	0.50–0.84	0.60	0.46–0.78	0.69	0.51–0.93
Age												
18–25			1.22	0.77–1.91	1.27	0.81–2.00	1.22	0.77–1.94	1.07	0.66–1.72	1.14	0.70–1.84
26–34			0.57	0.36–0.88	0.63	0.41–0.98	0.65	0.41–1.01	0.58	0.36–0.94	0.68	0.43–1.08
35–64			0.19	0.10–0.34	0.27	0.15–0.47	0.27	0.15–0.49	0.29	0.15–0.54	0.44	0.24–0.80
65+			…		…		…		…		…	
Education												
Less than high school			0.68	0.37–1.24	0.79	0.44–1.42	0.83	0.44–1.54	0.79	0.42–1.48	1.01	0.54–1.89
High school graduate			0.78	0.49–1.22	0.84	0.54–1.30	0.86	0.54–1.37	0.79	0.49–1.26	0.93	0.59–1.44
Some college			0.96	0.62–1.50	0.97	0.62–1.51	1.01	0.65–1.58	0.98	0.61–1.56	1.05	0.66–1.68
College graduate			…		…		…		…		…	
Employment												
Employed			…		…		…		…		…	
Unemployed			2.03	0.80–5.19	2.15	0.91–5.09	1.78	0.72–4.41	2.04	0.72–5.75	1.92	0.75–4.97
Not in labor force			1.03	0.72–1.47	1.10	0.79–1.55	1.05	0.74–1.50	1.06	0.72–1.55	1.18	0.81–1.71
Household income												
<$20,000			1.33	0.70–2.52	1.43	0.74–2.75	1.46	0.76–2.80	1.53	0.79–2.95	1.54	0.78–3.06
$20,000–39,99			1.28	0.84–1.95	1.35	0.88–2.07	1.41	0.92–2.16	1.45	0.92–2.28	1.55	0.99–2.43
$40,000–74,999			1.04	0.69–1.58	1.06	0.71–1.60	1.07	0.71–1.60	1.14	0.74–1.73	1.13	0.75–1.71
$75,000+			…		…		…		…		…	
Marital status												
Married			…		…		…		…		…	
Widowed/divorced/ separated			1.60	1.15–2.23	1.59	1.14–2.22	1.56	1.11–2.19	1.43	1.02–1.99	1.43	1.01–2.03
Never married			1.57	1.03–2.41	1.53	1.00–2.34	1.55	1.00–2.39	1.48	0.93–2.34	1.46	0.91–2.33
Health insurance coverage												
Yes			…		…		…		…		…	
No			1.89	0.98–3.62	1.92	0.98–3.76	1.79	0.94–3.40	2.00	1.06–3.79	2.18	1.11–4.28
Severe psychological distress												
Yes			1.80	1.28–2.54	1.71	1.21–2.41	1.63	1.12–2.39	1.57	1.09–2.26	1.49	1.02–2.17
No			…		…		…		…		…	
Urbanicity of residence												
Rural			0.64	0.40–1.03	0.59	0.36–0.96	0.66	0.41–1.05	0.68	0.42–1.11	0.64	0.39–1.04
Urban			…		…		…		…		…	
Difficult access to cannabis												
Yes					0.07	0.02–0.19					0.19	0.07–0.51
No					…						…	
Perception of risk of cannabis												
Yes							0.07	0.02–0.19			0.10	0.05–0.21
No							…				…	
Disapproval of cannabis use												
Yes									0.06	0.03–0.13	0.10	0.04–0.29
No									…		…	

Note. The tests of trends were conducted using the pooled sample. Model 1 included only survey year as a continuous variable; Model 2 included age, sex, education, employment status, household income, marital status, any health insurance coverage status, urbanicity of residence, year, and severe psychological distress. Model 3 added difficult access to cannabis to Model 2, Model 4 added perception of great risk of cannabis use to Model 2, and Model 5 added disapproval of cannabis use to Model 2. Model 6 added three cannabis-specific factors to Model 2. OR indicates odds ratios, and CI indicates 95% confidence intervals.
